# A real world analysis of COVID-19 impact on hospitalizations in older adults with chronic conditions from an Italian region

**DOI:** 10.1038/s41598-022-17941-2

**Published:** 2022-08-12

**Authors:** Cristina Bosetti, Magda Rognoni, Roberta Ciampichini, Luca Paroni, Marco Scala, Luca Cavalieri d’Oro, Alberto Zucchi, Andrea Amerio, Licia Iacoviello, Simone Ghislandi, Anna Odone, David Stuckler, Silvano Gallus, Lost in Lombardia Project Investigators, Cristina Bosetti, Silvano Gallus, Carlotta Micaela Jarach, Alessandra Lugo, Chiara Stival, Andrea Amerio, Mario Amore, Gianluca Serafini, Roberto De Sena, Simone Ghislandi, David Stuckler, Yuxi Wang, Marialaura Bonaccio, Francesco Gianfagna, Licia Iacoviello, Giansanto Mosconi, Anna Odone, Carlo Signorelli, Giacomo Vigezzi, Luca Cavalieri d’Oro, Luca Paroni, Marco Sala, Magda Rognoni, Roberta Ciampichini, Alberto Zucchi

**Affiliations:** 1 Department of Oncology, Laboratory of Methodology for Clinical Research, Istituto di Ricerche Farmacologiche Mario Negri IRCCS, Via Mario Negri 2, 20156 Milan, Italy; 2 Epidemiology Unit, Agenzia per la Tutela della Salute della Brianza, Monza, Italy; 3 Agenzia per la Tutela della Salute di Bergamo, Bergamo, Italy; 4 Department of Neuroscience, Rehabilitation, Ophthalmology, Genetics, Maternal and Child Health (DINOGMI), Section of Psychiatry, Università di Genova, Genoa, Italy; 5 IRCCS Ospedale Policlinico San Martino, Genoa, Italy; 6 Research Center in Epidemiology and Preventive Medicine (EPIMED), Department of Medicine and Surgery, University of Insubria, Varese, Italy; 7 Department of Epidemiology and Prevention, IRCCS Neuromed, Pozzilli, Italy; 8 Department of Social Sciences and Politics, Bocconi University, Milan, Italy; 9 School of Medicine, University Vita-Salute San Raffaele, Milan, Italy; 10 Department of Public Health, Experimental and Forensic Medicine, University of Pavia, Pavia, Italy; 11 Department of Environmental Health Sciences, Istituto di Ricerche Farmacologiche Mario Negri IRCCS, Milan, Italy

**Keywords:** Cancer, Cardiovascular diseases, Diabetes

## Abstract

Healthcare delivery reorganization during the COVID-19 emergency may have had a significant impact on access to care for older adults with chronic conditions. We investigated such impact among all adults with chronic conditions aged ≥ 65 years, identified through the electronic health databases of two local health agencies—ATS Brianza and ATS Bergamo—from the Lombardy region, Italy. We considered hospitalizations for 2020 compared to the average 2017–2019 and quantified differences using rate ratios (RRs). Overall, in 2017–2019 there were a mean of 374,855 older adults with  ≥ 1 chronic condition per year in the two ATS and 405,371 in 2020. Hospitalizations significantly decreased from 84,624 (225.8/1000) in 2017–2019 to 78,345 (193.3/1000) in 2020 (RR 0.86). Declines were reported in individuals with many chronic conditions and for most Major Diagnostic Categories, except for diseases of the respiratory system. The strongest reductions were observed in hospitalizations for individuals with active tumours, particularly for surgical ones. Hospitalization rates increased in individuals with diabetes, likely due to COVID-19-related diseases. Although determinants of the decrease in demand and supply for care among chronic older adults are to be further explored, this raises awareness on their impacts on chronic patients’ health in the medium and long run.

## Introduction

Since the end of January 2020, COVID-19—a disease caused by severe acute respiratory syndrome coronavirus 2 (SARS-CoV-2)—started to spread around the globe, and on March 11, 2020 the World Health Organization (WHO) declared a global pandemic^1,2^. In Italy, COVID-19 was first registered on February 21, 2020 in Codogno, Lombardy region, and it rapidly spread mainly in Northern Italy^3–5^. The Lombardy region was particularly hit by the pandemic, as it represents the Italian area with the highest COVID-19 incidence and death rates. In order to control the spread of COVID-19, from March 9 until May 4, 2020 a strict lockdown was imposed to the Italian population which limited all unnecessary activities^6^. By the end of September 2020—due to a second wave of COVID-19 infection—new lockdowns were ordered, with curfew and several gradients of restriction^7,8^.

Due to the high infection rate of SARS-CoV-2, the COVID-19 epidemic has placed strong pressure on the health care system and service delivery in Italy, as in most other countries around the globe^9,10^. Regional health services were reorganized, reducing access to outpatient and inpatient care only to urgent and essential situations and concentrating in specific hospital hubs the provision of health services for patients whose treatments could not be postponed^11,12^. Such measures may have significantly impacted on the population health, particularly for older adults with chronic conditions. Indeed, besides direct short-term health risks associated with SARS-CoV-2 infection due to their higher vulnerability to COVID-19 morbidity and mortality^13–17^, old chronic patients have been also at a higher risk of impaired health care, due to the limitations in the access to territorial and hospital health services they need during the COVID-19 outbreaks^18,19^. A few earlier studies have shown that emergency visits and hospitalizations decreased during the first waves of the COVID-19 pandemic in Italy^20,21^. A study conducted in seven hospitals from Italy showed that cancer diagnosis fell by 49% in 2020 compared with the average records of 2018–2019^22^. A retrospective study conducted in four hospitals from New York City during the pre, early, peak, and late COVID-19 epidemic found substantial decreases of non-COVID-19 hospitalizations in the peak COVID-19 period, the declines being observed across all diagnoses^23^. A retrospective analysis of national utilization data from Greece reported that hospital admissions and hospital surgical procedures significantly dropped by 17.3% and 24.8% respectively during the first 9 months of the epidemic compared to the average utilization rates of 2017–2019^24^.

To date, however, data on hospitalization patterns in individuals with chronic conditions are still scant at national and international level. With the present study, we aim to investigate and quantify the impact of the COVID-19 pandemic on the access to hospital services of older adults with chronic conditions using real-world data from the public health informative system of two large local health agencies from the Lombardy region, Italy.

## Results

Overall, there were an average of 374,855 individuals aged 65 years or more with a chronic condition per year in 2017–2019 and 405,371 in 2020. In 2020, 55.2% of individuals were women, mean age was 76.7 years (standard deviation 7.7), 46.9% of individuals were at low level of complexity and 5.4% at high level (Table 1). Most frequent chronic conditions were diabetes (10.2%), ischemic cardiopathy (7.4%), arrhythmic cardiomyopathy (5.7%), active tumours (5.7%), and heart failure (5.2%). Similar distributions were observed in 2017–2019. Moreover, the distribution of patients’ characteristics was comparable between the two ATS (Supplementary Table 1).

**Table 1:** Characteristics of individuals aged 65 years or more with a chronic disease from ATS Brianza and ATS Bergamo combined in 2017–2019 and 2020. Lombardy, Italy.

	N. patients (%)	
	Average 2017–2019	2020
Total	374,855	405,371
**Sex**		
Men	166,486 (44.4)	181,583 (44.8)
Women	208,369 (55.6)	223,788 (55.2)
**Age (years)**		
65–69	87,358 (23.3)	84,279 (20.8)
70–74	88,161 (23.5)	96,162 (23.7)
75–79	81,982 (21.9)	78,824 (19.4)
80–84	64,054 (17.1)	75,154 (18.5)
≥ 85	53,300 (14.2)	70,952 (17.5)
**Level of complexity**		
Low	175,758 (46.9)	190,222 (46.9)
Medium	178,840 (47.7)	193,274 (47.7)
High	20,257 (5.4)	21,875 (5.4)
**Chronic conditions**		
Diabetes	38,334 (10.2)	41,406 (10.2)
**Respiratory diseases**		
Asthma	1500 (0.4)	1755 (0.4)
COPD	10,108 (2.7)	10,790 (2.7)
**Tumours**		
Tumours, active	21,314 (5.7)	23,192 (5.7)
Tumours, follow-up	7079 (1.9)	7679 (1.9)
Tumours, remission	8356 (2.2)	8956 (2.2)
**Cardiovascular diseases**		
Ischemic cardiopathy	27,960 (7.5)	29,969 (7.4)
Valvular cardiopathy	5531 (1.5)	6123 (1.5)
Non-arrhythmic myocardiopathy	13,829 (3.7)	14,994 (3.7)
Arrhythmic myocardiopathy	21,648 (5.8)	23,202 (5.7)
Heart failure	19,831 (5.3)	21,082 (5.2)
Arterial vasculopathy	9756 (2.6)	10,323 (2.5)
Cerebral vasculopathy	13,667 (3.6)	14,304 (3.5)
**Renal diseases**		
Chronic renal failure	8454 (2.3)	9411 (2.3)
**Liver diseases**		
Hepatic cirrhosis	2145 (0.6)	2302 (0.6)
Chronic hepatitis	5513 (1.5)	6032 (1.5)
**Rheumatoid arthritis**	2534 (0.7)	3223 (0.8)
**Digestive diseases**		
Ulcerative colitis/Crohn	1322 (0.4)	1474 (0.4)
**Thyroid diseases**		
Hypothyroidism	3810 (1.0)	4649 (1.1)
Basedow’s disease/hyperthyroidism	376 (0.1)	464 (0.1)
Hashimoto thyroiditis	745 (0.2)	934 (0.2)

*COPD* chronic obstructive pulmonary disease.

Number and rates of hospitalizations in older adults with chronic conditions from the two ATS combined in 2017–2019 and 2020 are presented in Table 2. Overall hospitalizations significantly decreased from 84,624 (225.8/1000) in 2017–2019 to 78,345 (193.3/1000) in 2020, with a RR of 0.86. The strongest decreases were observed in day-hospital (RR = 0.71) and in surgical hospitalizations (RR = 0.72). With reference to level of complexity of chronic conditions, significant decreases were observed in both medium (RR = 0.83) and high level (RR = 0.62) hospitalizations. Results were very similar in the two ATS (Supplementary Table 2). Considering non-chronic individuals, we observed even stronger reduction in hospitalizations in 2020 as compared to 2017–2019 (RR = 0.69; Supplementary Table 3).

**Table 2:** Number of hospitalizations and corresponding rates (per 1000) among individuals aged 65 years or more with at least a chronic disease from ATS Brianza and ATS Bergamo combined, overall and by hospital regimen, type of hospitalization and level of complexity, in 2017–2019 and 2020, and rate ratio for 2020 versus 2017–2019, Lombardy, Italy.

	Average 2017–2019		2020		Rate ratio (95% CI)
	N	Rate	N	Rate	
Overall	84,624	225.8	78,345	193.3	0.86 (0.85–0.86)
**Hospital regimen**^a^					
Ordinary	72,565	193.6	68,149	168.1	0.87 (0.86–0.88)
Day-hospital	10,849	28.9	8357	20.6	0.71 (0.69–0.73)
**Type of diagnosis-related group**					
Medical	43,702	116.6	46,641	115.1	0.99 (0.97–1.00)
Surgical	40,912	109.1	31,704	78.2	0.72 (0.71–0.73)
**Level of complexity**					
Low	24,662	65.8	27,552	68.0	1.03 (1.02–1.05)
Medium	47,045	125.5	42,108	103.9	0.83 (0.82–0.84)
High	12,916	34.5	8685	21.4	0.62 (0.60–0.64)

*95% CI* 95% confidence interval.

^a^This category does not include sub-acute hospitalizations, included in overall hospitalizations.

Hospitalizations significantly decreased between 2017–2019 and 2020 in older adults with active tumours (RR = 0.47), ischemic cardiopathy (RR = 0.90), valvular cardiopathy (RR = 0.69), heart failure (RR = 0.76), arterial vasculopathy (RR = 0.63), cerebral vasculopathy (RR = 0.53), and chronic renal failure (RR = 0.71; Table 3). On the contrary, hospitalizations significantly increase in individuals with diabetes (RR = 1.30), chronic obstructive pulmonary disease (COPD; RR = 1.24), cancers in follow-up (RR = 1.38) and in remission (RR = 1.41), non-arrhythmic myocardiopathy (RR = 1.18), while no meaningful changes were found in individuals with other chronic conditions. Again, findings were consistent in the two ATS (Supplementary Table 4).

**Table 3:** Number of hospitalizations and corresponding rate (per 1000) among individuals aged 65 years or more with a chronic disease from ATS Brianza and ATS Bergamo by major chronic diseases in 2017–2019 and 2020, and rate ratio between the two periods. Lombardy, Italy.

	Average 2017–2019		2020		Rate ratio (95% CI)
	N	Rate	N	Rate	
Diabetes	5204	135.8	7337	177.2	1.30 (1.26–1.35)
**Respiratory diseases**					
Asthma	169	112.7	200	114.0	1.01 (0.82–1.24)
COPD	1643	162.5	2173	201.4	1.24 (1.16–1.32)
**Tumours**					
Tumours, active	16,156	758.0	8217	354.3	0.47 (0.46–0.48)
Tumours, follow-up	817	115.4	1227	159.8	1.38 (1.27–1.51)
Tumours, remission	734	87.8	1110	123.9	1.41 (1.29–1.55)
**Cardiovascular diseases**					
Ischemic cardiopathy	6637	237.4	6391	213.3	0.90 (0.87–0.93)
Valvular cardiopathy	1724	311.7	1324	216.2	0.69 (0.65–0.75)
Non arrhythmic myocardiopathy	1832	132.5	2345	156.4	1.18 (1.11–1.25)
Arrhythmic myocardiopathy	4872	225.1	5080	218.9	0.97 (0.93–1.01)
Heart failure	8052	406.0	6521	309.3	0.76 (0.74–0.79)
Arterial vasculopathy	5689	583.1	3813	369.4	0.63 (0.61–0.66)
Cerebral vasculopathy	5989	438.2	3328	232.7	0.53 (0.51–0.55)
**Renal diseases**					
Chronic renal failure	4211	498.1	3326	353.4	0.71 (0.68–0.74)
**Liver diseases**					
Hepatic cirrhosis	681	317.5	731	317.5	1.00 (0.90–1.11)
Chronic hepatitis	578	104.8	807	133.8	1.28 (1.15–1.42)
**Rheumatoid arthritis**	381	150.4	530	164.4	1.09 (0.96–1.25)
**Digestive diseases**					
Ulcerative colitis/Crohn	200	151.3	224	152.0	1.00 (0.83–1.22)
**Thyroid diseases**					
Hypothyroidism	340	89.2	404	86.9	0.97 (0.84–1.13)
Basedow’s disease/hyperthyroidism	24	63.8	36	77.6	1.22 (0.73–2.04)
Hashimoto thyroiditis	48	64.4	88	94.2	1.46 (1.03–2.08)

**Figure 1:**
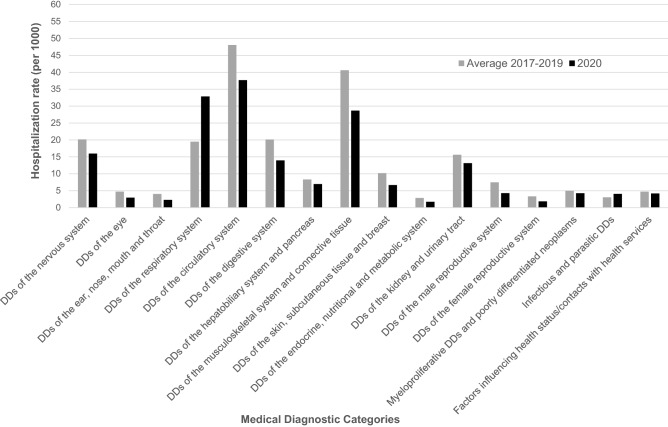
Hospitalization rates (per 1000) among individuals aged 65 years or more with a chronic disease from ATS Monza-Brianza and ATS Bergamo combined according to Major Diagnostic Categories in 2017–2019 and 2020. *DD* diseases and disorders.

When we analysed hospitalizations for individuals with active tumours according to type of hospitalization, we found that the reductions were greater for surgical (490.9/1000 vs 160.1/1000, RR = 0.33, 95% CI 0.31–0.34) than for medical hospitalizations (267.0/1000 vs 151.0/1000, RR = 0.57, 95% CI 0.54–0.59; data not shown). Hospitalization rates declined between 2017–2019 and 2020 for most MDCs, with the exception of diseases of the respiratory system, for which rates significantly increased (from 19.7/1000 to 34.3/1000, RR = 1.75; Fig. 1). Consistent patterns were found in ATS Brianza (Supplementary Fig. 1A) and ATS Bergamo (Supplementary Fig. 1B).

Considering individuals with diabetes, we found significant decreases for diseases of the musculoskeletal system and connective tissue (from 35.7/1000 to 27.0/1000, RR = 0.76, in ATS Brianza and from 43.1/1000 to 29.9/1000, RR = 0.69, in ATS Bergamo), while there were significant increases for other diseases, including in particular those of the nervous (from 6.8/1000 to 14.6/1000, RR = 2.15, and from 10.8/1000 to 16.1/1000, RR = 1.49), respiratory (from 9.3/1000 to 32.2/1000, RR = 3.46, and from 8.9 to 32.4/1000, RR = 3.64), and circulatory system (from 15.1/1000 to 31.5/1000, RR = 2.093, and from 16.5/1000 to 30.3/1000, RR = 1.84; Supplementary Fig. 2A,B). As a consequence, although the top most MDCs responsible for hospital admission among individuals with diabetes were similar in the two calendar periods, diseases of the respiratory system ranked 1° in 2020 in the two ATS compared to 5° in ATS Brianza and 6° in ATS Bergamo in 2017–2019 (Supplementary Table 5).

Among individuals with an active tumour, we found significant decreases for most MDCs, including in particular those of the skin, subcutaneous tissue, and breast (MDC 09), from 108.7/1000 to 16.0/1000, RR = 0.15 in ATS Brianza and from 156.8 to 22.7/1000, RR = 0.14 in ATS Bergamo) (Supplementary Fig. 3A,B). Among those individuals, diseases of the respiratory system ranked 1° in 2020 (they were 4° in ATS Brianza and 6° in ATS Bergamo in 2017–2019), while diseases of the skin, subcutaneous tissue, and breast ranked 9° and 7° respectively in ATS Brianza and ATS Bergamo in 2020, while they were 2° and 1° in 2017–2019; Supplementary Table 6).

## Discussion

In our study, we found that in Lombardy—one of the most heavily areas hit by COVID-19 pandemic in Italy, probably on account of its elderly population and the fact that was the first area to be reached by COVID-19 outside China—hospitalizations in older chronic patients have decreased by about 14% compared to the three previous years a consequence of the pressure the COVID-19 pandemic posed on health services. Such decreases were observed mainly for day-hospital hospitalizations and those involving surgical procedures. Consistent reductions in hospitalizations were found in individuals with many chronic conditions, but they were stronger in those with active tumours (by 53%), particularly when necessitating surgery (by 67%). We observed a decrease in hospitalizations for most MDCs, with the exception of those of the respiratory system, which are likely related to COVID-19. Considering the fact that we did not exclude hospitalizations due to COVID-19-related diagnoses, the reductions in hospital access during the 2020 COVID-19 pandemic could have been even larger in these populations. This notwithstanding, the declines in hospitalizations for chronic patients were more limited than those observed in non-chronic individuals.

Studies on the impact of COVID-19 emergency on hospitalization patterns in individuals with chronic conditions are scanty. A few earlier studies investigated hospitalization for acute (mainly cardiovascular) conditions and access to emergency departments, reporting significant reductions in hospitalization for acute myocardial infarction, stroke, heart failure, and other cardiovascular diseases^20,21,23,25–31^.

Consistently, in our study we found that individuals with various cardiovascular conditions, including ischemic and valvular cardiopathy, heart failure, and arterial and cerebral vasculopathy, had a reduced access to hospital during 2020. This notwithstanding the fact that individuals with cardiovascular diseases—as those with many other chronic conditions—may have been at an increased risk of hospitalization for COVID-19^15,17^.

To the best of our knowledge no study investigated the impact of COVID-19 in individuals with diabetes^32^. We found an approximately 30% increase in hospitalizations for individuals with diabetes. This appears to be largely due to the rises in hospitalizations due to diseases of the nervous, respiratory, and circulatory system, which are likely attributable to COVID-19. Patients diabetes have indeed been reported to be among those at a high risk of more severe SARS-CoV-2 infection requiring hospitalization, due to the frequent presence of other concomitant disease^15,17^. The significant reduction in hospitalizations for diseases of the skin, musculoskeletal and connective tissue, on the contrary, can be due to a more frequent treatment at home during the COVID-19 emergency of diabetes-related complications included in MDC 09.

A few previous studies, mainly conducted in a single centre and on specific cancer sites, reported a reduction in hospital access in cancer patients during 2020^30^. Our study confirms that hospitalizations for individuals with an active tumour, particularly those involving surgery, were strongly reduced during the COVID-19 pandemic in 2020. In cancer patients, hospitalizations declined particularly for diseases in MDC 09 which include malignancies of the breast. Our data underline the difficulty in providing optimal health care in cancer patients, that appear to be among those that suffered the most for the lack of necessary cure during the COVID-19 crisis^33^. The reduction in hospitalizations for cancer patients may have significant long-term effects on morbidity and mortality in those patients. It has indeed been estimated that even modest delays in surgery for cancer will determine a significant impact on survival^34^, and a few studies already reported an increase in out-of-hospital mortality mainly driven by deaths for neoplasms, cardiovascular and endocrine diseases^20^, although there will need some more time to quantify the exact extent of such impact.

With reference to respiratory diseases, we found an increase in hospitalizations for COPD but not for asthma. The increases in hospitalization of patients with COPD are likely due to the higher frequency of severe relapse of the illness and also of potentially COVID-19-related diseases, which have been shown to be at increased risk of SARS-CoV-2 infection^15,17^. However, a few previous studies which could analyse non-COVID-19 related hospitalizations for acute asthma and other respiratory diseases reported significant reductions, which have been partly attributed to a reduced exposure to air pollutants and other respiratory infections during the COVID-19 lockdowns^23,26,28,30^.

Among other chronic conditions, we reported a reduction by 30% in hospitalizations among patients with chronic renal failure. This is consistent with findings from a recent meta-analysis indicating that the COVID-19 pandemic led to reductions in access to kidney transplantation, dialysis, and in-person nephrology care, with an overall a 50% reduction in nephrology-related hospital admissions reported in four studies on a total of 4873 patients^35^.

There are two major explanations of the observed decrease in hospitalizations for many chronic patients, the first linked the reduction of health service offer, due to the restrictions in hospital services, with the reallocation of several hospital departments and personnel to the care of COVID-19 patients and the consequent cancellations of elective, routine, and non-urgent treatments, and the second related to the limited patients’ access, due to the patient’s fear of SARS-CoV-2 infection which kept them away from hospital, as well as from other health care services^26,31,35^. It is also possible that underlining trends in hospitalization explain some of our findings, although we did not find any meaningful trends in hospitalizations for chronic patients over the four calendar years considered.

Our study has the strength to be a large real-world, population-based study conducted on all older adults with chronic diseases in the study areas (including over 2.3 million older adults and 400,000 chronic patients), while most previous studies were conducted in single or few centres, and were focused on specific conditions. Most previous studies, moreover, were conducted on acute conditions, while very few analysed accesses to hospital among chronic patients. Furthermore, we could compare two populations from northern Italy—both particularly hit by COVID-19 during the first and second waves—which provided extremely consistent results. Among the major limitations of the study, there is the fact that we could not distinguish COVID-19-related hospitalizations, since at the time of our analyses we had no information on hospitalizations for this cause. Considering that hospitalizations primarily due to COVID-19 have subsequently been estimated to be approximatively 7% in our population, the reductions in hospital access during the 2020 COVID-19 pandemic could have been even larger. Moreover, we could not analyse other outcomes, as cause-specific mortality rates, because no data on cause of death were available in our data.

In conclusion, the present study provides evidence of a significantly reduced hospital care for older adults with chronic conditions during the COVID-19 pandemic. This was noted in particular for those with cancer and necessitating surgery, suggesting that those patients may not have been receiving adequate health services and treatments since the pandemic began, with likely detrimental health consequences on the medium and long run. However, the declines in hospitalization rates were more modest than those observed in non-chronic patients, indicating that during the outbreaks of COVID-19 in 2020 there some attempt to foster the cure for these more vulnerable individuals.

## Methods

We conducted an observational study on all older adults with chronic conditions identified from the electronic health databases of ATS (Agenzia per la Tutela della Salute) Brianza and ATS Bergamo, Lombardy, Italy. Each ATS has a central role in programming, paying, and evaluating health services to approximately 2.3 million residents, including about 400,000 individuals with chronic conditions.

Health data available in the two ATS include the following information for all residents: demographic data (age, sex, area of residence, and vital status); exemptions from payment for specific diseases, including chronic ones; and detailed discharge information from public and private accredited hospitals (date of admission and discharge, main diagnosis and five secondary diagnoses, date and type of intervention, and costs associated with hospitalization on the basis of the weight of Diagnosis-Related Group—DRG—system)^36,37^. The information available for each individual in the various databases are identified by the same unique code and were subsequently linked with a deterministic process in order to identify individuals of interest and their related information.

The study population consisted of all adults aged 65 or more living in the two areas covered by ATS Brianza and ATS Bergamo over the years between 2017 and 2020, and who had at least one chronic disease. For all inhabitants in Lombardy, it was searched evidence for chronic conditions on the basis of data from various databases of the Health Informative System. On a hierarchical basis, individuals were classified according to their primary chronic condition using algorithms defined by the Lombardy region for the year 2017^38^. The conditions were then classified into nine major disease categories, i.e., diabetes mellitus, respiratory diseases, tumours, cardiovascular diseases, renal diseases, liver diseases, rheumatoid arthritis, digestive diseases, and thyroid diseases. Tumours were also considered in three into separate group, i.e., active (less than 1 year since diagnosis), in follow-up (1–5 years), and in remission (more than 5 years).

The study was conducted in accordance with the Declaration of Helsinki and its protocol was approved by the Ethical Committees of the two ATS (Comitato Etico Brianza, Protocol N. 3599, and Comitato Etico della Provincia di Bergamo, Protocol N. 3599 69/21). The Ethical Committees waived the requirement for informed consent for the collection, analysis, and publication of the retrospectively and anonymized data used in this study. Personnel of the two ATS had the had the appropriate permissions to access patient data from the databases.

As indicators of access to health care, we considered numbers and rates of hospitalization; these were provided overall, by hospital regimen (ordinary, day-hospital), type of DRG (medical, surgical), level of complexity (low, 1 comorbidity; medium, 2–3 comorbidities; high, ≥ 3 comorbidities), individuals’ chronic conditions, and Major Diagnostic Category (MDC)^37^. Rates were computed as the ratio between number of hospital discharges over 1 year divided by the referent population multiplied by 1000. Numbers and rates of hospitalization are presented for the average period 2017–2019 and the year 2020. Differences in hospitalization between the two periods were compared using the rate ratios (RRs) and their corresponding 95% confidence intervals (CIs). For a comparative purpose, hospitalizations among non-chronic individuals were also assessed. A yearly ranking system has been defined based on the MDC prevalence for individuals with diabetes (with and without complications) and for individuals with active tumours^39^.

Data analyses were performed using the software SAS, version 9.4 (SAS Institute, North Carolina, USA) and Stata, version 17 (StataCorp, College Station, Texas 77845, USA).

## Supplementary Information

Supplementary Information.

## Data Availability

Data that support the findings of this study and materials are available from the corresponding author upon request.
