# Supplementary material for: A real world analysis of COVID-19 impact on hospitalizations in older adults with chronic conditions from an Italian region
Source: Sci Rep. 2022 Aug 12;12:13704. doi: 10.1038/s41598-022-17941-2 (PMC9374749; doi:10.1038/s41598-022-17941-2)
Supplement: Supplementary file 1 — Supplementary Information. [file 41598_2022_17941_MOESM1_ESM.docx]

**Supplementary Table 1.** Characteristics of individuals aged 65 years or more with a chronic disease from ATS Brianza and ATS Bergamo in 2017-2019 and 2020. Lombardy, Italy.

|  | **ATS Brianza**  **N. Patients (%)** | | **ATS Bergamo**  **N. Patients (%)** | |
| --- | --- | --- | --- | --- |
|  | **Average**  **2017-2019** | **2020** | **Average**  **2017-2019** | **2020** |
|  |  |  |  |  |
| Total | 201402 | 222385 | 173453 | 182986 |
| Sex |  |  |  |  |
| Men | 89168 (44.3) | 99425 (44.7) | 77318 (44.6) | 82158 (44.9) |
| Women | 112235 (55.7) | 122960 (55.3) | 96134 (55.4) | 100828 (55.1) |
| Age (years) |  |  |  |  |
| 65-69 | 46291 (23.0) | 45128 (20.3) | 41056 (23.7) | 39151 (21.4) |
| 70-74 | 47389 (23.5) | 51730 (23.3) | 40777 (23.5) | 44432 (24.3) |
| 75-79 | 44456 (22.1) | 43184 (19.4) | 37531 (21.6) | 35640 (19.5) |
| 80-84 | 35057 (17.4) | 42345 (19.0) | 28987 (16.7) | 32809 (17.9) |
| ≥85 | 28209 (14.0) | 39998 (18.0) | 25101 (14.5) | 30954 (16.9) |
| Level of complexity |  |  |  |  |
| Low | 94308 (46.8) | 103397 (46.5) | 81450 (47.0) | 86825 (47.4) |
| Medium | 96146 (47.7) | 106610 (47.9) | 82694 (47.7) | 86664 (47.4) |
| High | 10948 (5.4) | 12378 (5.6) | 9309 (5.4) | 9497 (5.2) |
| Chronic conditions |  |  |  |  |
| Diabetes | 20199 (10.0) | 22241 (10.0) | 18135 (10.5) | 19165 (10.5) |
| Respiratory diseases |  |  |  |  |
| Asthma | 944 (0.5) | 1103 (0.5) | 556 (0.3) | 652 (0.4) |
| COPD | 5077 (2.5) | 5534 (2.5) | 5031 (2.9) | 5256 (2.9) |
| Tumours |  |  |  |  |
| Tumours, active | 11120 (5.5) | 12498 (5.6) | 10194 (5.9) | 10694 (5.8) |
| Tumours, follow-up | 3503 (1.7) | 3844 (1.7) | 3576 (2.1) | 3835 (2.1) |
| Tumours, remission | 4266 (2.1) | 4710 (2.1) | 4090 (2.4) | 4246 (2.3) |
| Cardiovascular diseases |  |  |  |  |
| Ischemic cardiopathy | 14840 (7.4) | 16262 (7.3) | 13120 (7.6) | 13707 (7.5) |
| Valvular cardiopathy | 2889 (1.4) | 3225 (1.5) | 2642 (1.5) | 2898 (1.6) |
| Non-arrhythmic myocardiopathy | 7523 (3.7) | 8345 (3.8) | 6306 (3.6) | 6649 (3.6) |
| Arrhythmic myocardiopathy | 11105 (5.5) | 12162 (5.5) | 10543 (6.1) | 11040 (6.0) |
| Heart failure | 10982 (5.5) | 12175 (5.5) | 8849 (5.1) | 8907 (4.9) |
| Arterial vasculopathy | 5109 (2.5) | 5648 (2.5) | 4647 (2.7) | 4675 (2.6) |
| Cerebral vasculopathy | 7464 (3.7) | 8000 (3.6) | 6203 (3.6) | 6304 (3.4) |
| Renal diseases |  |  |  |  |
| Chronic renal failure | 4906 (2.4) | 5705 (2.6) | 3548 (2.0) | 3706 (2.0) |
| Liver diseases |  |  |  |  |
| Hepatic cirrhosis | 1098 (0.5) | 1250 (0.6) | 1047 (0.6) | 1052 (0.6) |
| Chronic hepatitis | 2043 (1.0) | 2263 (1.0) | 3470 (2.0) | 3769 (2.1) |
| Rheumatoid arthritis | 1174 (0.6) | 1480 (0.7) | 1360 (0.8) | 1743 (1.0) |
| Digestive diseases |  |  |  |  |
| Ulcerative colitis/Crohn | 808 (0.4) | 916 (0.4) | 514 (0.3) | 558 (0.3) |
| Thyroid diseases |  |  |  |  |
| Hypothyroidism | 2098 (1.0) | 2587 (1.2) | 1712 (1.0) | 2062 (1.1) |
| Basedow’s disease/hyperthyroidism | 206 (0.1) | 244 (0.1) | 170 (0.1) | 220 (0.1) |
| Hashimoto thyroiditis | 298 (0.1) | 363 (0.2) | 447 (0.3) | 571 (0.3) |
|  |  |  |  |  |

COPD: chronic obstructive pulmonary disease.

**Supplementary Table 2.** Number of hospitalizations and corresponding rates (per 1000) among individuals aged 65 years or more with a chronic disease from ATS Brianza and ATS Bergamo, overall and by hospital regimen and type of hospitalization, in 2017-2019 and 2020, and rate ratio between the two periods, Lombardy, Italy.

|  | **Hospitalizations ATS Brianza** | | | | | **Hospitalizations ATS Bergamo** | | | | |
| --- | --- | --- | --- | --- | --- | --- | --- | --- | --- | --- |
|  | **Average**  **2017-2019** | | **2020** | | **Rate ratio**  **(95% CI)** | **Average**  **2017-2019** | | **2020** | | **Rate ratio**  **(95% CI)** |
|  | **N.** | **Rate** | **N.** | **Rate** |  | **N.** | **Rate** | **N.** | **Rate** |  |
|  |  |  |  |  |  |  |  |  |  |  |
| Overall | 41644 | 206.8 | 40640 | 182.8 | 0.88 (0.87-0.90) | 42980 | 247.8 | 37705 | 206.1 | 0.83 (0.82-0.84) |
| Hospital regimen^a^ |  |  |  |  |  |  |  |  |  |  |
| Ordinary | 36269 | 180.1 | 35931 | 161.6 | 0.90 (0.88-0.91) | 36296 | 209.3 | 32218 | 176.1 | 0.84 (0.82-0.85) |
| Day-hospital | 4855 | 24.1 | 3693 | 16.6 | 0.69 (0.66-0.72) | 5994 | 34.6 | 4664 | 25.5 | 0.74 (0.71-0.77) |
| Type of diagnosis-related group |  |  |  |  |  |  |  |  |  |  |
| Medical | 21012 | 104.3 | 24175 | 108.7 | 1.04 (1.02-1.06) | 22690 | 130.8 | 22466 | 122.8 | 0.94 (0.92-0.96) |
| Surgical | 20622 | 102.4 | 16465 | 74.0 | 0.72 (0.71-0.74) | 20290 | 117.0 | 15239 | 83.3 | 0.71 (0.70-0.73) |
| Level of complexity |  |  |  |  |  |  |  |  |  |  |
| Low | 11886 | 59.0 | 13999 | 62.9 | 1.07 (1.04-1.09) | 12776 | 73.7 | 13553 | 74.1 | 1.01 (0.98-1.03) |
| Medium | 23130 | 114.8 | 21879 | 98.4 | 0.86 (0.84-0.87) | 23915 | 137.9 | 20229 | 110.5 | 0.80 (0.79-0.82) |
| High | 6628 | 32.9 | 4762 | 21.4 | 0.65 (0.63-0.68) | 6288 | 36.3 | 3923 | 21.4 | 0.59 (0.57-0.61) |
|  |  |  |  |  |  |  |  |  |  |  |

95% CI: 95% confidence interval; ^a^This category does not include sub-acute hospitalizations, included in overall hospitalizations.

**Supplementary Table 3.** Number of hospitalizations and corresponding rates (per 1000) among individuals aged 65 years without chronic diseases from ATS Brianza and ATS Bergamo combined, overall and by hospital regimen and type of hospitalization, in 2017-2019 and 2020, and rate ratio between the two periods, Lombardy, Italy.

|  | **Average**  **2017-2019** | | **2020** | | **Rate ratio**  **(95% CI)** |
| --- | --- | --- | --- | --- | --- |
|  | **N.** | **Rate** | **N.** | **Rate** |  |
|  |  |  |  |  |  |
| Overall | 34241 | 217.3 | 20747 | 149.2 | 0.69 (0.67-0.70) |
| Hospital regimen^a^ |  |  |  |  |  |
| Ordinary | 30503 | 193.5 | 18508 | 133.1 | 0.69 (0.68-0.70) |
| Day-hospital | 2569 | 16.3 | 1785 | 12.8 | 0.79 (0.74-0.83) |
| Type of diagnosis-related group |  |  |  |  |  |
| Medical | 24148 | 153.2 | 13870 | 99.8 | 0.65 (0.64-0.67) |
| Surgical | 10089 | 64 | 6877 | 49.5 | 0.77 (0.75-0.80) |
|  |  |  |  |  |  |

95% CI: 95% confidence interval.

^a^This category does not include sub-acute hospitalizations, included in overall hospitalizations.

**Supplementary Table 4.** Number of hospitalizations and corresponding rate (x 1000 patients) among individuals aged 65 years or more with a chronic disease from ATS Brianza and ATS Bergamo by major chronic diseases in 2017-2019 and 2020. and rate ratio between the two periods. Lombardy. Italy.

|  | **Hospitalizations ATS Brianza** | | | | | **Hospitalizations ATS Bergamo** | | | | |
| --- | --- | --- | --- | --- | --- | --- | --- | --- | --- | --- |
|  | **Average**  **2017-2019** | | **2020** | | **Rate ratio**  **(95% CI)** | **Average**  **2017-2019** | | **2020** | | **Rate ratio**  **(95% CI)** |
|  | **N.** | **Rate** | **N.** | **Rate** |  | **N.** | **Rate** | **N.** | **Rate** |  |
|  |  |  |  |  |  |  |  |  |  |  |
| Diabetes | 2499 | 123.7 | 3827 | 172.1 | 1.39 (1.32-1.46) | 2705 | 149.2 | 3510 | 183.1 | 1.23 (1.17-1.29) |
| Respiratory diseases |  |  |  |  |  |  |  |  |  |  |
| Asthma | 104 | 110.2 | 119 | 107.9 | 0.98 (0.75-1.27) | 65 | 117.6 | 81 | 124.2 | 1.06 (0.76-1.50) |
| COPD | 819 | 161.3 | 1080 | 195.2 | 1.21 (1.11-1.33) | 824 | 163.9 | 1093 | 208.0 | 1.27 (1.16-1.39) |
| Tumours |  |  |  |  |  |  |  |  |  |  |
| Tumours. active | 7778 | 699.5 | 4224 | 338.0 | 0.48 (0.47-0.50) | 8378 | 821.8 | 3993 | 373.4 | 0.45 (0.44-0.47) |
| Tumours. follow-up | 379 | 108.2 | 577 | 150.1 | 1.39 (1.22-1.58) | 438 | 122.6 | 650 | 169.5 | 1.38 (1.22-1.57) |
| Tumours. remission | 365 | 85.6 | 548 | 116.3 | 1.36 (1.19-1.55) | 369 | 90.2 | 562 | 132.4 | 1.47 (1.28-1.68) |
| Cardiovascular diseases |  |  |  |  |  |  |  |  |  |  |
| Ischemic cardiopathy | 3218 | 216.9 | 3242 | 199.4 | 0.92 (0.88-0.97) | 3419 | 260.6 | 3149 | 229.7 | 0.88 (0.84-0.93) |
| Valvular cardiopathy | 850 | 294.2 | 639 | 198.1 | 0.67 (0.61-0.75) | 874 | 331.0 | 685 | 236.4 | 0.71 (0.66-0.79) |
| Non arrhythmic myocardiopathy | 915 | 121.6 | 1261 | 151.1 | 1.24 (1.14-1.35) | 917 | 145.4 | 1084 | 163.0 | 1.12 (1.03-1.23) |
| Arrhythmic myocardiopathy | 2356 | 212.2 | 2528 | 207.9 | 0.98 (0.93-1.04) | 2516 | 238.6 | 2552 | 231.2 | 0.97 (0.92-1.02) |
| Heart failure | 3981 | 362.5 | 3520 | 289.1 | 0.80 (0.76-0.83) | 4071 | 460.0 | 3001 | 336.9 | 0.73 (0.69-0.76) |
| Arterial vasculopathy | 2850 | 557.8 | 1944 | 344.2 | 0.62 (0.58-0.65) | 2839 | 610.9 | 1869 | 399.8 | 0.65 (0.62-0.69) |
| Cerebral vasculopathy | 3027 | 405.5 | 1713 | 214.1 | 0.53 (0.50-0.56) | 2962 | 477.5 | 1615 | 256.2 | 0.54 (0.50-0.57) |
| Renal diseases |  |  |  |  |  |  |  |  |  |  |
| Chronic renal failure | 2242 | 457.0 | 1950 | 341.8 | 0.75 (0.70-0.79) | 1969 | 555.0 | 1376 | 371.3 | 0.67 (0.62-0.72) |
| Liver diseases |  |  |  |  |  |  |  |  |  |  |
| Hepatic cirrhosis | 305 | 277.8 | 398 | 318.4 | 1.15 (0.99-1.33) | 376 | 359.2 | 333 | 316.5 | 0.88 (0.76-1.02) |
| Chronic hepatitis | 185 | 90.6 | 270 | 119.3 | 1.32 (1.09-1.59) | 393 | 113.2 | 537 | 142.5 | 1.26 (1.10-1.44) |
| Rheumatoid arthritis | 167 | 142.2 | 241 | 162.8 | 1.14 (0.94-1.39) | 214 | 157.1 | 289 | 165.8 | 1.06 (0.89-1.26) |
| Digestive diseases |  |  |  |  |  |  |  |  |  |  |
| Ulcerative colitis/Crohn | 114 | 141.1 | 147 | 160.5 | 1.14 (0.89-1.45) | 86 | 167.9 | 77 | 138.0 | 0.82 (0.60-1.13) |
| Thyroid diseases |  |  |  |  |  |  |  |  |  |  |
| Hypothyroidism | 178 | 84.8 | 185 | 71.5 | 0.84 (0.69-1.04) | 162 | 94.6 | 219 | 106.2 | 1.12 (0.91-1.38) |
| Basedow’s disease /hyperthyroidism | 14 | 68.0 | 18 | 73.8 | 1.09 (0.54-2.18) | 10 | 60.9 | 18 | 81.8 | 1.34 (0.61-3.37) |
| Hashimoto thyroiditis | 12 | 40.3 | 25 | 68.9 | 1.71 (0.86-3.40) | 36 | 81.2 | 63 | 110.3 | 1.36 (0.90-2.12) |
|  |  |  |  |  |  |  |  |  |  |  |

**Supplementary Table 5.** Ranking of Major Diagnostic Categories (MDC) categories among individuals aged 65 years or more with diabetes from ATS Brianza and ATS Bergamo in 2019 and 2020. Lombardy. Italy.

| **MDC code** | **MDC description** | **Average**  **2017-2019** | **2020** |
| --- | --- | --- | --- |
| **ATS Brianza** | |  |  |
| 4 | Diseases and disorders of the respiratory system | 5 | 1 |
| 5 | Diseases and disorders of the circulatory system | 2 | 2 |
| 8 | Diseases and disorders of the musculoskeletal system and connective tissue | 1 | 3 |
| 1 | Diseases and disorders of the nervous system | 6 | 4 |
| 11 | Diseases and disorders of the kidney and urinary tract | 4 | 5 |
| 6 | Diseases and disorders of the digestive system | 3 | 6 |
| 7 | Diseases and disorders of the hepatobiliary system and pancreas | 7 | 7 |
| 9 | Diseases and disorders of the skin. subcutaneous tissue and breast | 12 | 8 |
| 18 | Infectious and Parasitic DDs (Systemic or unspecified sites) | 13 | 9 |
| 12 | Diseases and disorders of the male reproductive system | 9 | 10 |
| **ATS Bergamo** | |  |  |
| 4 | Diseases and disorders of the respiratory system | 6 | 1 |
| 5 | Diseases and disorders of the circulatory system | 2 | 2 |
| 8 | Diseases and disorders of the musculoskeletal system and connective tissue | 1 | 3 |
| 1 | Diseases and disorders of the nervous system | 4 | 4 |
| 6 | Diseases and disorders of the digestive system | 3 | 5 |
| 11 | Diseases and disorders of the kidney and urinary tract | 5 | 6 |
| 9 | Diseases and disorders of the skin. subcutaneous tissue and breast | 12 | 7 |
| 7 | Diseases and disorders of the hepatobiliary system and pancreas | 9 | 8 |
| 23 | factors influencing health status and other contacts with health services | 11 | 9 |
| 12 | Diseases and disorders of the male reproductive system | 9 | 10 |

**Supplementary Table 6.** Ranking of Major Diagnostic Categories (MDC) categories among individuals aged 65 years or more with active tumours from ATS Brianza and ATS Bergamo in 2019 and 2020. Lombardy. Italy.

| **MDC code** | **MDC description** | **Average 2017-2019** | **2020** |
| --- | --- | --- | --- |
| **ATS Brianza** | |  |  |
| 4 | Diseases and disorders of the respiratory system | 4 | 1 |
| 11 | Diseases and disorders of the kidney and urinary tract | 1 | 2 |
| 5 | Diseases and disorders of the circulatory system | 7 | 3 |
| 6 | Diseases and disorders of the digestive system | 3 | 4 |
| 8 | Diseases and disorders of the musculoskeletal system and connective tissue | 9 | 5 |
| 17 | Myeloproliferative DDs (poorly differentiated neoplasms) | 5 | 6 |
| 7 | Diseases and disorders of the hepatobiliary system and pancreas | 8 | 7 |
| 1 | Diseases and disorders of the nervous system | 10 | 8 |
| 9 | Diseases and disorders of the skin. subcutaneous tissue and breast | 2 | 9 |
| 18 | Infectious and parasitic DDs (systemic or unspecified sites) | 15 | 10 |
| **ATS Bergamo** | |  |  |
| 4 | Diseases and disorders of the respiratory system | 6 | 1 |
| 11 | Diseases and disorders of the kidney and urinary tract | 2 | 2 |
| 5 | Diseases and disorders of the circulatory system | 7 | 3 |
| 17 | Myeloproliferative DDs (poorly differentiated neoplasms) | 5 | 4 |
| 8 | Diseases and disorders of the Musculoskeletal system and connective tissue | 8 | 5 |
| 6 | Diseases and disorders of the digestive system | 3 | 6 |
| 9 | Diseases and disorders of the skin, subcutaneous tissue and breast | 1 | 7 |
| 1 | Diseases and disorders of the nervous system | 11 | 8 |
| 23 | Factors influencing health status and other contacts with health services | 10 | 9 |
| 7 | Diseases and disorders of the hepatobiliary system and pancreas | 9 | 10 |

DD: diseases and disorders.

**A**

**B**

**Supplementary Figure 1.** Hospitalization rates (per 1000) among individuals aged 65 years or more with a chronic disease from ATS Brianza (A) and ATS Bergamo (B) according to Major Diagnostic Categories in 2017-2019 and 2020. Lombardy. Italy.

DD: diseases and disorders.

**A**

**B**

**Supplementary Figure 2.** Hospitalizations rates (per 1000) among individuals aged 65 years or more with diabetes from ATS Brianza (A) and ATS Bergamo (B) according to Major Diagnostic Categories. Lombardy. Italy. 2017-2020.

DD: diseases and disorders.

**A**

**B**

**Supplementary Figure 3.** Hospitalizations rates (per 1000) among individuals aged 65 years or more with active tumours from ATS Brianza (A) and ATS Bergamo (B) according to Major Diagnostic Categories. Lombardy. Italy. 2017-2020.

DD: diseases and disorders.
